# 

**Published:** 1889-12

**Authors:** 


CHRISTMAS GREETING.—The JOURNAL extends the right hand to all its readers, and bids them "Shake." Here we are, by God's Providence, come around to the close of another year,—to the blessed Christmas, that era of rejoicing in all Christian nations; and we heartily wish all a "Merry Christmas"! May peace and plenty, health, happiness and honor be the portion of all who have given aid or comfort to this enterprise, which like a small seed, fell by the wayside, took root, sprung into life, and nurtured by kindly interest and liberal patronage, has developed into full flower, shedding its perfume throughout the world.

